# S06-1: Collaborating across diverse clinical areas and locations to tackle deconditioning in the acute healthcare setting #ActiveWards

**DOI:** 10.1093/eurpub/ckae114.222

**Published:** 2024-09-26

**Authors:** Juliet Harvey

**Affiliations:** University of Dundee

## Abstract

**Purpose:**

To provide opportunity to reduce deconditioning in the clinical in-patient environment by using person-centred practice development methods to empower staff to be agents of change in their local specialty area.

**Project Description:**

In the acute hospital setting deconditioning is a major risk factor with around 90% of the day spent sitting/lying. In this setting we have the opportunity to promote physical activity and reduce sedentary behaviour. As experts in activity and movement, Physiotherapists and Occupational Therapists routinely assess function/activity of patients and provide support from brief intervention through to complex rehabilitation, behaviour change interventions and lifestyle prescription, as well as signposting or referring on to other services. The therapists work in multi-disciplinary teams to provide person-centred care to the patient in the treatment of injury, illness, or disability. To scope the situation a survey was conducted resulting in the formation of a Special Interest Group (SIG). The purpose of the group was to provide peer-support and act as a platform for sharing resources and ideas relating to patient activity. Group members (N = 23) were from across NHS Greater Glasgow and Clyde including Older Peoples Services, Stroke, Medical, Surgical, Orthopaedics, Oncology, Critical Care, Neurology and Physical Disability Teams.

The SIG provided a collaborative hub where staff networked, supported each other, conducted projects, and shared their findings and challenges. Members worked with staff groups, patients, carers, health improvement, medical illustrations, digital, patient experience, equality and diversity teams, volunteer services, third sector organisations and international consensus groups. The outputs from the group were shared widely (#ActiveWards) and included development of resources, audit tools and training material. The group formed Active Wards Principles, which are now included in board-wide guidelines and standards.

**Conclusion:**

Tackling deconditioning in the clinical environment requires complex interventions that are tailored to the patient, teams, and local circumstance. The work demonstrates what can be achieved when staff are provided with a little time and given ownership of change processes. There are now >50 members in the group. The work has had a ripple effect across our health board and to other organisations.

